# Ecosystem services and agriculture: tradeoffs and synergies

**DOI:** 10.1098/rstb.2010.0143

**Published:** 2010-09-27

**Authors:** Alison G. Power

**Affiliations:** Department of Ecology and Evolutionary Biology, Cornell University, Ithaca, NY, USA

**Keywords:** ecosystem services, agroecosystems, pollination, biological control, valuation of ecosystem services, soil carbon sequestration

## Abstract

Agricultural ecosystems provide humans with food, forage, bioenergy and pharmaceuticals and are essential to human wellbeing. These systems rely on ecosystem services provided by natural ecosystems, including pollination, biological pest control, maintenance of soil structure and fertility, nutrient cycling and hydrological services. Preliminary assessments indicate that the value of these ecosystem services to agriculture is enormous and often underappreciated. Agroecosystems also produce a variety of ecosystem services, such as regulation of soil and water quality, carbon sequestration, support for biodiversity and cultural services. Depending on management practices, agriculture can also be the source of numerous disservices, including loss of wildlife habitat, nutrient runoff, sedimentation of waterways, greenhouse gas emissions, and pesticide poisoning of humans and non-target species. The tradeoffs that may occur between provisioning services and other ecosystem services and disservices should be evaluated in terms of spatial scale, temporal scale and reversibility. As more effective methods for valuing ecosystem services become available, the potential for ‘win–win’ scenarios increases. Under all scenarios, appropriate agricultural management practices are critical to realizing the benefits of ecosystem services and reducing disservices from agricultural activities.

## 1. Introduction

**Figure 1.**
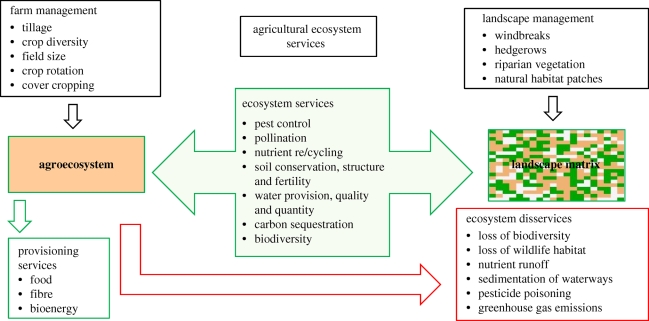
Impacts of farm management and landscape management on the flow of ecosystem services and disservices to and from agroecosystems.

Agriculture is a dominant form of land management globally, and agricultural ecosystems cover nearly 40 per cent of the terrestrial surface of the Earth (FAO 2009). Agroecosystems are both providers and consumers of ecosystem services (figure 1). Humans value these systems chiefly for their provisioning services, and these highly managed ecosystems are designed to provide food, forage, fibre, bioenergy and pharmaceuticals. In turn, agroecosystems depend strongly on a suite of ecosystem services provided by natural, unmanaged ecosystems. Supporting services include genetic biodiversity for use in breeding crops and livestock, soil formation and structure, soil fertility, nutrient cycling and the provision of water. Regulating services may be provided to agriculture by pollinators and natural enemies that move into agroecosystems from natural vegetation. Natural ecosystems may also purify water and regulate its flow into agricultural systems, providing sufficient quantities at the appropriate time for plant growth.

Traditionally, agroecosystems have been considered primarily as sources of provisioning services, but more recently their contributions to other types of ecosystem services have been recognized (MEA 2005). Influenced by human management, ecosystem processes within agricultural systems can provide services that support the provisioning services, including pollination, pest control, genetic diversity for future agricultural use, soil retention, regulation of soil fertility and nutrient cycling. Whether any particular agricultural system provides such services in support of provisioning depends on management, and management is influenced by the balance between short-term and long-term benefits.

Management practices also influence the potential for ‘disservices’ from agriculture, including loss of habitat for conserving biodiversity, nutrient runoff, sedimentation of waterways, and pesticide poisoning of humans and non-target species (Zhang *et al*. 2007). Since agricultural practices can harm biodiversity through multiple pathways, agriculture is often considered anathema to conservation. However, appropriate management can ameliorate many of the negative impacts of agriculture, while largely maintaining provisioning services.

Agroecosystems can provide a range of other regulating and cultural services to human communities, in addition to provisioning services and services in support of provisioning. Regulating services from agriculture may include flood control, water quality control, carbon storage and climate regulation through greenhouse gas emissions, disease regulation, and waste treatment (e.g. nutrients, pesticides). Cultural services may include scenic beauty, education, recreation and tourism, as well as traditional use. Agricultural places or products are often used in traditional rituals and customs that bond human communities. Conservation of biodiversity may also be considered a cultural ecosystem service influenced by agriculture, since most cultures recognize appreciation of nature as an explicit human value. In return, biodiversity can contribute a variety of supporting services to agroecosystems and surrounding ecosystems (Daily 1997).

Around the world, agricultural ecosystems show tremendous variation in structure and function, because they were designed by diverse cultures under diverse socioeconomic conditions in diverse climatic regions. Functioning agroecosystems include, among others, annual crop monocultures, temperate perennial orchards, grazing systems, arid-land pastoral systems, tropical shifting cultivation systems, smallholder mixed cropping systems, paddy rice systems, tropical plantations (e.g. oil palm, coffee, cacao), agroforestry systems and species-rich home gardens. This variety of agricultural systems results in a highly variable assortment and quantity of ecosystem services. Just as the provisioning services and products that derive from these agroecosystems vary, the support services, regulating services and cultural services also differ, resulting in extreme variation in the value these services provide, inside and outside the agroecosystem. In maximizing the value of provisioning services, agricultural activities are likely to modify or diminish the ecological services provided by unmanaged terrestrial ecosystems, but appropriate management of key processes may improve the ability of agroecosystems to provide a broad range of ecosystem services.

Globally, most landscapes have been modified by agricultural activities and most natural, unmanaged ecosystems sit in a matrix of agricultural land uses. The conversion of undisturbed natural ecosystems to agriculture can have strong impacts on the system's ability to produce important ecosystem services, but many agricultural systems can also be important sources of services. Indeed, agricultural land use can be considered an intermediate stage in a human-impact continuum between wilderness and urban ecosystems (Swinton *et al*. 2007). Just as conversion from natural ecosystems to agriculture can reduce the flow of certain ecosystem services, the intensification of agriculture (Matson *et al*. 1997) or the conversion of agroecosystems to urban or suburban development can further degrade the provision of beneficial services.

## 2. Approaches to analysing ecosystem services

The value of ecosystem services has been estimated in various ways. In general, the framework has three main parts: (i) measuring the provision of ecosystem services; (ii) determining the monetary value of ecosystem services; (iii) designing policy tools for managing ecosystem services (Polasky 2008). Ecologists and other natural scientists have been engaged in enhancing our understanding of how ecosystem services are produced for over a decade (e.g. Costanza *et al*. 1997; Daily 1997; MEA 2005). Basic knowledge about ecosystem structure and function is increasing at a rapid pace, but we know less about how these factors determine the provision of a complete range of ecosystem services from an individual ecosystem (NRC 2005). In practice, most studies focus on estimating the provision of one or two well understood ecosystem services. Better understanding of the processes that influence ecosystem services could allow us to predict the outputs of a range of ecosystem services, given particular ecosystem characteristics and perturbations to those ecosystems. That is, an ‘ecological production function’ might be generated (Polasky 2008). Despite recent advances, this is an area of research that still needs considerable attention.

The second step of valuation of ecosystem services typically includes both market and non-market valuation. Valuing the provisioning services that derive from agriculture is relatively straightforward, since agricultural commodities are traded in local, regional or global markets. Some ecosystem services provide an essential input to agricultural production, and their value can be measured by estimating the change in the quantity or quality of agricultural production when the services are removed or degraded. This approach has been used to estimate the value of pollination services and biological control services (e.g. Losey & Vaughan 2006; Gallai *et al*. 2009). Values for such services can also be estimated by measuring replacement costs, such as pesticides replacing natural pest control and hand-pollination or beehive rental replacing pollination.

Non-market valuation methods have been used for many years to measure both the use value and the non-use value of various environmental amenities (Mendelsohn & Olmstead 2009). Non-market valuation can be based on revealed preference (behaviour expressed through consumer choices) or stated preference (e.g. attitudes expressed through surveys). In contingent valuation surveys, for example, consumers are asked what they would be willing to pay for the ecosystem service. Another approach is to ask producers—in this case farmers—what they would be willing to accept to supply the ecosystem service (Swinton *et al*. 2007).

The overarching goal of measuring and valuing ecosystem services is to use that information to shape policies and incentives for better management of ecosystems and natural resources. One of the inherent difficulties of managing ecosystem services is that the individuals who control the supply of such services, such as farmers and other land managers, are not always the beneficiaries of these services. Many ecosystem services are public goods. While farmers do benefit from a variety of ecosystem services, their activities may strongly influence the delivery of services to other individuals who do not control the production of these services. Examples include the impact of farming practices on downstream water supply and purity and regional pest management. The challenge is to use emerging information about ecological production functions and valuation to develop policies and incentives that are easily implemented and adaptable to changing ecological and market conditions.

One approach to incentives is to provide payments for environmental services, through government programmes or private sector initiatives (Swinton 2008). Historically, the US has provided support for soil conservation investments and other readily observable practices to maintain or enhance certain ecosystem services. In the US, the Conservation Security Program of the 2002 farm bill established payments for environmental services, and many European countries have also provided governmental support for environmentally sound farming practices that support ecosystem services. Agri-environment schemes are intended to moderate the negative environmental effects of intensive agriculture by providing financial incentives to farmers to adopt environmentally sound agricultural practices. The impacts of these projects are variable, however, and their success is debated (e.g. Baulcombe *et al*. 2009). A recent evaluation of over 200 paired fields in five European countries indicated that agri-environment programmes had marginal to moderate positive impacts on biodiversity, but largely failed to benefit rare or endangered species (Kleijn *et al*. 2006).

The Economics of Ecosystems and Biodiversity (TEEB) led by the United Nations Environment Programme (UNEP), is an international effort designed to integrate science, economics and policy around biodiversity and ecosystem services. A recent report for policy-makers highlights the link between poverty and the loss of ecosystems and biodiversity, with the intent of facilitating the development of effective policy in this area (ten Brink 2009). Another approach is the establishment of markets for pollution credits, including the growing global carbon market operating under various cap and trade initiatives, such as the European Union Emission Trading System.

## 3. Ecosystem services flowing to agriculture

The production of agricultural goods is highly dependent on the services provided by neighbouring natural ecosystems, but only recently have there been attempts to estimate the value of many of those services to agricultural enterprises. Some services are more easily quantified than others, to the extent that they are essential to crop production or they substitute directly for purchased inputs.

### (a) Biological pest control

Biological control of pest insects in agroecosystems is an important ecosystem service that is often supported by natural ecosystems. Non-crop habitats provide the habitat and diverse food resources required for arthropod predators and parasitoids, insectivorous birds and bats, and microbial pathogens that act as natural enemies to agricultural pests and provide biological control services in agroecosystems (Tscharntke *et al*. 2005). These biological control services can reduce populations of pest insects and weeds in agriculture, thereby reducing the need for pesticides.

Because the ecosystem services provided by natural enemies can substitute directly for insecticides and crop losses to pests can often be measured, the economic value of these services is more easily estimated than many other services. For example, an analysis of the value of natural enemy suppression of soya bean aphid in soya bean indicated that this ecosystem service was worth a minimum of US$239 million in four US states in 2007–2008 alone (Landis *et al*. 2008). Since this is an estimate of the value of suppressing a single pest in one crop, the total value of biological control services is clearly much larger. Natural pest control services have been estimated to save $13.6 billion per year in agricultural crops in the US (Losey & Vaughan 2006). This estimate is based on the value of crop losses to insect damage as well as the value of expenditures on insecticides. Studies suggest that insect predators and parasitoids account for approximately 33 per cent of natural pest control (Hawkins *et al*. 1999), therefore the value of pest control services attributed to insect natural enemies has been estimated at $4.5 billion per year (Losey & Vaughan 2006).

### (b) Pollination

Pollination is another important ecosystem service to agriculture that is provided by natural habitats in agricultural landscapes. Approximately 65 per cent of plant species require pollination by animals, and an analysis of data from 200 countries indicated that 75 per cent of crop species of global significance for food production rely on animal pollination, primarily by insects (Klein *et al*. 2007). Of the most important animal-pollinated crops, over 40 per cent depend on wild pollinators, often in addition to domesticated honeybees. Only 35–40% of the total volume of food crop production comes from animal-pollinated crops, however, since cereal crops typically do not depend on animal pollination. Aizen *et al*. (2009) used data from the United Nations Food and Agriculture Organization (FAO) on the production of 87 globally important crops during 1961–2006 to estimate that the consequences of a complete loss of pollinators for total global agricultural production would be a reduction of 3–8%. The percentage increase in total cultivated area that would be required to compensate for the decrease in production was much higher, particularly in the developing world where agriculture is more pollinator-dependent.

Like biological control, pollination services are more readily quantified than many other services. Early estimates of the value of pollination services were based on the total value of animal-pollinated crops, but recent estimates have been more nuanced. Since most crops are only partly dependent on animal pollination, a dependence ratio or a measure of the proportion reduction in production in the absence of pollinators can provide a better approximation of production losses in the absence of pollinators (Gallai *et al*. 2009). Clearly, these estimates are also fairly crude and intended to provide a broad-brush assessment of potential economic benefits. Moreover, most estimates do not take into account potential changes in the value of each commodity as demand increases owing to reduced crop production.

A recent assessment of agricultural vulnerability to loss of pollination services based on the ratio of the economic value of insect pollination to the economic value of the crop indicated an overall vulnerability of 9.5 per cent, but vulnerability varied significantly among types of commodities as well as by geographical region (Gallai *et al*. 2009). Stimulant crops (coffee, cacao, and tea), nuts, fruits and edible oil crops were predicted to be particularly vulnerable to the loss of pollination services (table 1). The economic impact of insect pollination on world food production in 2005 in the 162 FAO member countries has been calculated at 153 billion euro, but vulnerability to loss of pollinators varies among geographical regions due, in part, to crop specialization (Gallai *et al*. 2009). For example, West African countries produce 56 per cent of the world's stimulant crops with a vulnerability to pollinator loss of 90 per cent. The loss of pollination services in these crops could have devastating effects on the economies of such countries in the short term and lead to significant restructuring of global prices in the longer term (Gallai *et al*. 2009).

**Table 1.** Rate of vulnerability to pollinator loss and effect of pollinator loss on global food production for pollinator-dependent crop categories based on 2005 data. IPEV, insect pollination economic value; EV, total production economic value. Adapted from Gallai *et al*. (2009).

crop category	rate of vulnerability (IPEV/EV) %	relative production surplus^a^ (% of consumption)	
		before pollinator loss	after pollinator loss
stimulant crops	39.0	18	−24
nuts	31.0	29	16
fruits	23.1	12	−12
edible oil crops	16.3	75	40
vegetables	12.2	19	−6
pulse	4.3	60	54
spices	2.7	11	8

^a^The difference between 2005 production and consumption expressed in relative terms as % of 2005 consumption figures following FAO (http://faostat.fao.org).

A crucial question is whether the loss of pollination services could jeopardize world food supply. Gallai *et al*. (2009) conclude that overall production would keep pace with consumption, but a complete loss of pollinators would cause global deficits in fruits, vegetables and stimulants (table 1). Such declines in production could result in significant market disruptions as well as nutrient deficiencies, even if total caloric intake is still sufficient.

### (c) Water quantity and quality

The provision of sufficient quantities of clean water is an essential ecological service provided to agroecosystems, and agriculture accounts for about 70 per cent of global water use (FAO 2003). Perennial vegetation in natural ecosystems such as forests can regulate the capture, infiltration, retention and flow of water across the landscape. The plant community plays a central role in regulating water flow by retaining soil, modifying soil structure and producing litter. Forest soils tend to have a higher infiltration rate than other soils, and forests tend to reduce peak flows and floods while maintaining base flows (Maes *et al*. 2009). Through hydraulic lift and vertical uplifting, deep rooting species can improve the availability of both water and nutrients to other species in the ecosystem. In addition, soil erosion rates are usually low, resulting in good water quality. Fast-growing plantation forests may be an exception to this generalization, however; they can help regulate groundwater recharge, but they may reduce stream flow and salinize or acidify some soils (Jackson *et al*. 2005).

Water availability in agroecosystems depends not only on infiltration and flow, but also on soil moisture retention, another type of ecosystem service. While the supply of surface water and groundwater (‘blue water’) inputs to agriculture through irrigation are indispensable in some parts of the world, 80 per cent of agricultural water use comes from rainfall stored in soil moisture (‘green water’; Molden 2007). Water storage in soil is regulated by plant cover, soil organic matter and the soil biotic community (bacteria, fungi, earthworms, etc.). Trapping of sediments and erosion are controlled by the architecture of plants at or below the soil surface, the amount of surface litter and litter decomposition rate. Invertebrates that move between the soil and litter layer influence water movement within soil, as well as the relative amounts of infiltration and runoff (Swift *et al*. 2004). These soil processes provide essential ecosystem services to agriculture.

With climate change, increased variability of rainfall is predicted to lead to greater risk of drought and flood, while higher temperatures will increase water demand (IPCC 2007). Estimates of water availability for agriculture often neglect the contribution of green water, but predictions about water availability in 2050 are highly dependent on the inclusion of green water. Whereas more than six billion people are predicted to experience water shortages in 2050 when only blue water is taken into account, this number drops to about four billion when both blue and green water availability is taken into account (Rockström *et al*. 2009). Some regions of the world are much more dependent on green water than others (Rockström *et al*. 2009).

On-farm management practices that target green water can significantly alter these predictions of water shortages (Rost *et al*. 2009). For example, modifying the tillage regime or mulching can reduce soil evaporation by 35–50%. Rainwater harvest and on-farm storage in ponds, dykes or subsurface dams can allow farmers to redirect water to crops during periods of water stress, recovering up to 50 per cent of water normally lost to the system. By incorporating moderate values (25%) for reductions in soil evaporation and water harvesting into a dynamic global vegetation and water balance model, Rost *et al*. (2009) predicted that global crop production could be increased by nearly 20 per cent, a value comparable to the current contribution of irrigation, from on-farm green water management practices.

True markets for water are rare (Mendelsohn & Olmstead 2009), and the value of hydrological ecosystem services to agriculture is only partially accounted for in most estimates. Most farmers who withdraw surface waters directly do not pay for these services, except where local water sources are controlled by irrigation districts. Agricultural water demand estimates are often based on production data, where the marginal value of water is estimated by the increase in profits from a unit increase in water inputs. Production data can be highly variable, however, and increases in production can be difficult to assign to water inputs (Mendelsohn & Olmstead 2009). Although market approaches for direct water pricing are available, they tend to focus on blue water in a particular water basin. Many water prices for agricultural use are based on groundwater removal, using the energy costs of pumping as the key input variable. The relatively new approach of payments for environmental services has often focused on supporting watershed protection and water quality enhancements that target the provision of blue water (Wunder *et al*. 2008). It has been suggested recently that farmers should receive payments or ‘green water credits’ from downstream water users for good management practices that enhance green water retention as well as blue water conservation (ISRIC 2007).

### (d) Soil structure and fertility

Soil structure and fertility provide essential ecosystem services to agroecosystems (Zhang *et al*. 2007). Well-aerated soils with abundant organic matter are fundamental to nutrient acquisition by crops, as well as water retention. Soil pore structure, soil aggregation and decomposition of organic matter are influenced by the activities of bacteria, fungi and macrofauna, such as earthworms, termites and other invertebrates. Micro-organisms mediate nutrient availability through decomposition of detritus and plant residues and through nitrogen fixation. Agricultural management practices that degrade soil structure and soil microbial communities include mechanical ploughing, disking, cultivating and harvesting, but management practices can also protect the soil and reduce erosion and runoff. Conservation tillage and other soil conservation measures can maintain soil fertility by minimizing the loss of nutrients and keeping them available to crops. Cover crops facilitate on-farm retention of soil and nutrients between crop cycles, while hedgerows and riparian vegetation reduce erosion and runoff among fields. Incorporation of crop residues can maintain soil organic matter, which assists in water retention and nutrient provision to crops. Together these practices conserve a suite of ecosystem services to agriculture from the soil.

### (e) Landscape influences on the delivery of ecosystem services to agriculture

The delivery of ecosystem services to agriculture is highly dependent on the structure of the landscape in which the agroecosystem is embedded (figure 1). Agricultural landscapes span a continuum from structurally simple landscapes dominated by one or two cropping systems to complex mosaics of diverse cropping systems embedded in a natural habitat matrix. Water delivery to agroecosystems depends on flow patterns across the landscape and can be influenced by a variety of biophysical factors. Stream flow is influenced by withdrawals for irrigation, as well as landscape simplification. Water provisioning is also affected by diversion to other uses in the landscape or watershed, such as domestic, industrial or energy consumption.

Both natural biological control services and pollination services depend crucially on the movement of organisms across the agricultural landscape, and hence the spatial structure of the landscape strongly influences the magnitude of these ecological services to agricultural ecosystems (Tscharntke *et al*. 2005; Kremen *et al*. 2007). In complex landscapes, natural enemies and pollinators move among natural and semi-natural habitats that provide them with refugia and resources that may be scarce in crop fields (Coll 2009). Natural enemies with the ability to disperse long distances or that have large home ranges are better able to survive in disturbed agricultural landscapes with fewer or more distant patches of natural habitat (Tscharntke *et al*. 2005).

Agricultural intensification can jeopardize many of the ecosystem services provided by the landscape (Matson *et al*. 1997). Across large areas of North America and Western Europe, agricultural intensification has resulted in a simplification of landscape structure through the expansion of agricultural land, increase in field size, loss of field margin vegetation and elimination of natural habitat (Robinson & Sutherland 2002). This simplification tends to lead to higher levels of pest damage and lower populations of natural enemies (Brewer *et al*. 2008; Gardiner *et al*. 2009; O'Rourke 2010). A meta-analysis of the effects of landscape structure on natural enemies and pests in agriculture showed that landscape complexity enhanced natural enemy populations in 74 per cent of cases, whereas pest pressure was reduced in more complex landscapes in 45 per cent of cases (Bianchi *et al*. 2006). Natural enemies such as predators and parasitoids appear to respond to landscape structure at smaller spatial scales than herbivorous insects (Brewer *et al*. 2008; O'Rourke 2010) and may be more susceptible to habitat fragmentation. Based on a review of 16 studies of nine crops on four continents, Klein *et al*. (2007) concluded that agricultural intensification threatens wild bee communities and hence may degrade their stabilizing effect on pollination services at the landscape level. Recent studies have suggested that farm-level diversification is more likely to influence pests and natural enemies if the wider landscape is structurally simple, than if it is already very complex (Tscharntke *et al*. 2005; O'Rourke 2010). In complex landscapes, adding farm-level complexity does not necessarily enhance the benefits of pest control services.

Agricultural intensification in the landscape can diminish other ecosystem services as well. Protection of groundwater and surface water quality can be threatened by intensification because of increased nutrients, agrochemicals and dissolved salts (Dale & Polasky 2007). Loss of riparian vegetation that often accompanies intensification can result in significant sedimentation of waterways and dams. Other studies, however, have suggested that initial conversion to agriculture can cause significant reductions in ecosystem services, but subsequent intensification of the system may not have large impacts (Steffan-Dewenter *et al*. 2007). Since the quantification of intensification can be highly variable among studies and agricultural systems, these results may not be incompatible. The bulk of evidence indicates that increasing agricultural intensification will erode many ecosystem services, and projections indicate that 80 per cent of crop production growth in developing countries through to 2030 will come through intensification (FAO 2006).

Not all agricultural landscapes are currently shaped by intensification. Interestingly, changes in agricultural policies that encourage regional specialization have led to intensification in some European landscapes, accompanied by cropland abandonment in others (Stoate *et al*. 2009). Widespread abandonment of agricultural land without restoration presents its own set of problems, including landscape degradation, increased risk of erosion and fire. In some areas, both agricultural intensification and land abandonment coexist in the same landscapes, and both processes may influence the delivery of ecosystem services to agroecosystems (Stoate *et al*. 2009).

## 4. Ecosystem services and disservices from agriculture

Agroecosystems are essential sources of provisioning services, and the value of the products they provide are readily measured using standard market analysis. Depending on their structure and management, they may also contribute a number of other ecosystem services (MEA 2005). Ecosystem processes operating within agricultural systems can provide some of the same supporting services described above, including pollination, pest control, genetic diversity for future agricultural use, soil retention, and regulation of soil fertility, nutrient cycling and water. In addition, agricultural systems can be managed to support biodiversity and enhance carbon sequestration—globally important ecosystem services.

### (a) Ecosystem disservices from agriculture

Agriculture can contribute to ecosystem services, but can also be a source of disservices, including loss of biodiversity, agrochemical contamination and sedimentation of waterways, pesticide poisoning of non-target organisms, and emissions of greenhouse gases and pollutants (Dale & Polasky 2007; Zhang *et al*. 2007). These disservices come at a significant cost to humans, but there is often a mismatch between the benefits, which accrue to the agricultural sector, and the costs, which are typically borne by society at various scales, from local communities impacted by pesticides in drinking water to the global commons affected by global warming. Linking these disservices more closely to agricultural activities through incorporating the externalities into the costs of production has the potential to reduce these negative environmental consequences of agricultural practices.

#### (i) Nutrient cycling and pollution

From the local scale to the global scale, agriculture has profound effects on biogeochemical cycles and nutrient availability in ecosystems (Vitousek *et al*. 1997; Galloway *et al*. 2004). The two nutrients that most limit biological production in natural and agricultural ecosystems are nitrogen and phosphorus, and they are also heavily applied in agroecosystems. Nitrogen and phosphorus fertilizers have greatly increased the amount of new nitrogen and phosphorus in the biosphere and have had complex, often harmful, effects on natural ecosystems (Vitousek *et al*. 1997). These anthropogenically mobilized nutrients have entered both groundwater and surface waters, resulting in many negative consequences for human health and the environment. Approximately 20 per cent of N fertilizer applied in agricultural systems moves into aquatic ecosystems (Galloway *et al*. 2004). Impacts of nutrient loss from agroecosystems include groundwater pollution and increased nitrate levels in drinking water, eutrophication, increased frequency and severity of algal blooms, hypoxia and fish kills, and ‘dead zones’ in coastal marine ecosystems (Bouwman *et al*. 2009).

Ecosystem services within agroecosystems can be supported by nutrient management strategies that recouple nitrogen, phosphorus and carbon cycling within the agroecosystem. Under conventional practice in developed countries, agroecosystems are often maintained in a state of nutrient saturation and are inherently leaky as a result of chronic surplus additions of nitrogen and phosphorus (Galloway *et al*. 2004; Drinkwater & Snapp 2007; Vitousek *et al*. 2009). In developing countries, soils are more likely to be depleted and nutrients may be much more limiting to production, though chronic nutrient surpluses may still occur in some systems (table 2; Vitousek *et al*. 2009).

**Table 2.** Inputs and outputs of nitrogen and phosphorus in three corn cropping systems with similar yield potential: a low-input corn-based system in western Kenya; a highly fertilized wheat-corn double-cropping system in north China; and a corn–soya bean rotation in IL, USA. Actual yields of corn were 2000, 8500 and 8200 kg ha^−1^ yr^−1^ per crop in the Kenya, China and USA systems, respectively; the Chinese and USA systems also yielded wheat and soya bean, respectively, in a separate cropping season. From Vitousek *et al*. (2009).

inputs and outputs	nutrient balances by region (kg ha^−1^ yr^−1^)					
	western Kenya		north China		midwest USA	
	N	P	N	P	N	P
fertilizer	7	8	588	92	93	14
biological N fixation					62	
total agronomic inputs	7	8	588	92	155	14
removal in grain and/or beans	23	4	361	39	145	23
removal in other harvested products	36	3				
total agronomic outputs	59	7	361	39	145	23
agronomic inputs minus harvest removals	−52	+1	+227	+53	+10	−9

To maintain ecosystem services, soil nutrient pools can be intentionally managed to supply crops at the right time, while minimizing nutrient losses by reducing soluble inorganic nitrogen and phosphorus pools (Drinkwater & Snapp 2007). Practices such as cover cropping or intercropping enhance plant and microbial assimilation of nitrogen and reduce standing pools of nitrate, the form of nitrogen that is most susceptible to loss. Other good management practices include diversifying nutrient sources, legume intensification for biological nitrogen fixation and phosphorus-solubilizing properties, and diversifying rotations. Integrated management of biogeochemical processes that regulate the cycling of nutrients and carbon could reduce the need for surplus nutrient additions in agriculture (Drinkwater & Snapp 2007).

Recent analyses forecasting human alterations of soil nitrogen and phosphorus cycling under various scenarios to 2050 further emphasize that closing nutrient cycles in agroecosystems can significantly influence soil nutrient balance (Bouwman *et al*. 2009). Spatially explicit modelling of soil nitrogen and phosphorus balances suggest that soil phosphorus will be depleted in grasslands around the world and rock phosphate reserves will be reduced by 36–64% by 2100. Many scenarios indicate increases in soil nitrogen over this period along with increased leaching and denitrification losses, though nitrogen balances are likely to decline in North American and Europe because of ongoing changes in management practices (Bouwman *et al*. 2009).

Other ecosystem disservices from agriculture include applications of pesticides that result in loss of biodiversity and pesticide residues in surface and groundwater, which degrades the water provisioning services provided by agroecosystems. Moreover, agriculture modifies the species identity and root structure of the plant community, the production of litter, the extent and timing of plant cover and the composition of the soil biotic community, all of which influence water infiltration and retention in the soil. The intensity of agricultural production and management practices affect both the quantity and quality of water in an agricultural landscape. Practices that maximize plant cover, such as minimum tillage, polycultures or agroforestry systems are likely to decrease runoff and increase infiltration. Irrigation practices also influence runoff, sedimentation and groundwater levels in the landscape.

#### (ii) Emissions of greenhouse gases

Agricultural activities are estimated to be responsible for 12–14% of global anthropogenic emissions of greenhouse gases, not including emissions that arise from land clearing (US-EPA 2006; IPCC 2007). After fossil fuel combustion, land-use change is the second largest global cause of CO_2_ emissions, and some of this change is driven by conversion to agriculture, largely in developing countries. In developed countries, forest conversion to cropland, pasture and rangeland were common through the middle of the twentieth century, but current conversions are primarily for suburban development. In addition to losses of above-ground carbon due to deforestation or other land clearing, conversion of natural ecosystems to agriculture reduces the soil carbon pool by 30–50% over 50–100 years in temperate regions and 50–75% over 20–50 years in the tropics (Lal 2008*a*). Although agricultural systems generate very large CO_2_ fluxes to and from the atmosphere, the net flux appears to be small. However, both the magnitude of emissions and the relative importance of the different sources vary widely among agricultural systems around the world.

Agricultural activities contribute to emissions in several ways (table 3). Approximately 49 per cent of global anthropogenic emissions of methane (CH_4_) and 66 per cent of global annual emissions of nitrous oxide (N_2_O), both greenhouse gases, are attributed to agriculture (FAO 2003), although there is a wide range of uncertainty in the estimates of both the agricultural contribution and the anthropogenic total. N_2_O emissions occur naturally as a part of the soil nitrogen cycle, but the application of nitrogen to crops can significantly increase the rate of emissions, particularly when more nitrogen is applied than can be taken up by the plants. Nitrogen is added to soils through the use of inorganic fertilizers, application of animal manure, cultivation of nitrogen-fixing plants and retention of crop residues. Globally, approximately 50 per cent of N applied as fertilizer is taken up by the crop, 2–5% is stored as soil N, 25 per cent is lost as N_2_O emissions and 20 per cent moves to aquatic systems (Galloway *et al*. 2004). In addition to direct N_2_O emissions, the production of synthetic nitrogen fertilizers is an energy-intensive process that produces additional greenhouse gases. Flooded rice cultivation contributes to greenhouse gas emissions through anaerobic decomposition of soil organic matter by CH_4_-emitting soil microbes. The practice of burning crop residues contributes to the production of both CH_4_ and N_2_O.

**Table 3.** Agricultural contributions to global greenhouse gas emissions by source and expected changes in agricultural greenhouse gas emissions by 2030. Adapted from FAO (2003).

greenhouse gas	CO_2_ carbon dioxide	CH_4_ methane	N_2_O nitrous oxide	NO_*x*_ nitric oxides	ammonia
agricultural source (estimated % contribution to total emissions)^a^	land use change, especially deforestation	ruminants (15%)	livestock/manure (17%)	biomass burning (13%)	livestock/manure (44%)
		rice (11%)	mineral fertilizers (8%)	manure/mineral fertilizers (2%)	mineral fertilizers (17%)
		biomass burning (7%)	biomass burning (3%)		biomass burning (11%)
agricultural emissions (as % total of anthropogenic sources)	15%	49%	66%	27%	93%
expected changes in agricultural emissions by 2030	stable or decreasing	rice—stable or decreasing	35–60% increase		from livestock—60% increase
		livestock—60% increase			

^a^Total emissions include both natural and anthropogenic sources.

Livestock production also contributes to CH_4_ and N_2_O emissions (Pitesky *et al*. 2009), and these impacts are likely to increase through to 2050 as the demand for meat increases (FAO 2003). Ruminant livestock such as cattle, sheep, goats and buffalo emit CH_4_ as a byproduct of their digestive processes (enteric fermentation). Livestock waste can release both CH_4_, through the biological breakdown of organic compounds, and N_2_O, through microbial metabolism of nitrogen contained in manure. The magnitude of direct emissions depends strongly on manure management practices, such as the use of lagoons or field spreading, and to some degree on the type of livestock feed. The magnitude of emissions attributed to livestock is controversial, ranging from 3 to 18 per cent of global emissions, depending on whether the effects of land-clearing (i.e. deforestation) for livestock production is included in the estimate (Pitesky *et al*. 2009).

### (b) Ecosystem services from agriculture

On-farm management practices can significantly enhance the ecosystem services provided by agriculture. Farmers routinely manage for greater provisioning services by using inputs and practices to increase yields, but management practices can also enhance other ecosystem services, such as pollination, biological pest control, soil fertility and structure, water regulation, and support for biodiversity. Habitat management within the agroecosystem can provide the resources necessary for pollinators or natural enemies (Tscharntke *et al*. 2005). Many studies have identified the important role of perennial vegetation in supporting biodiversity in general and beneficial organisms in particular (e.g. Perfecto & Vandermeer 2008). Evidence suggests that management systems that emphasize crop diversity through the use of polycultures, cover crops, crop rotations and agroforestry can often reduce the abundance of insect pests that specialize on a particular crop, while providing refuge and alternative prey for natural enemies (Andow 1991). Similar practices may benefit wild pollinators, including minimal use of pesticides, no-till systems and crop rotations with mass-flowering crops.

#### (i) Mitigation of greenhouse gases emissions

Agricultural practices can effectively reduce or offset agricultural greenhouse gas emissions through a variety of processes (Drinkwater & Snapp 2007; Lal 2008*a*; Smith *et al*. 2008). Effective manure management can significantly reduce emissions from animal waste. Replacing synthetic nitrogen fertilizers with biological nitrogen fixation by legumes can reduce CO_2_ emissions from agricultural production by half (Drinkwater & Snapp 2007). The process of perennialization and legume intensification in agroecosystems modifies internal cycling processes and increases N use efficiency within agroecosystems via the recoupling mechanisms discussed above. Chronic surplus additions of inorganic N, which are currently commonplace, can be reduced under these scenarios, leading to reductions in NO_*x*_ and N_2_O emissions.

Agriculture can offset greenhouse gas emissions by increasing the capacity for carbon uptake and storage in soils, i.e. carbon sequestration (Lal 2008*a*,*b*). The net flux of CO_2_ between the land and the atmosphere is a balance between carbon losses from land-use conversion and land-management practices, and carbon gains from plant growth and sequestration of decomposed plant residues in soils. In particular, soil conservation measures such as conservation tillage and no-till cultivation can conserve soil carbon, and crop rotations and cover crops can reduce the degradation of subsurface carbon. In general, water management and erosion control can aid in maintaining soil organic carbon (Lal 2008*a*).

Soil carbon sequestration thus provides additional ecosystem services to agriculture itself, by conserving soil structure and fertility, improving soil quality, increasing the use efficiency of agronomic inputs, and improving water quality by filtration and denaturing of pollutants (Lal 2008*b*; Smith *et al*. 2008). The economic benefits of conservation agriculture have been estimated in diverse systems around the world, from smallholder agricultural systems in Latin America and sub-Saharan Africa to large-scale commercial production systems in Brazil and Canada (reviewed in Govaerts *et al*. 2009). Many farmers have already adopted practices that retain soil C in order to achieve higher productivity and lower costs. However, even the use of soil conservation and restoration practices cannot fully restore soil carbon lost through conversion to agriculture. It is estimated that the soil C pool attainable through best management practices is typically 60–70% of the original soil C pool prior to conversion (Lal 2008*a*).

Finally, agricultural land can also be used to grow crops for bioenergy production. Bioenergy, particularly cellulosic biofuels, has the potential to replace a portion of fossil fuels and to lower greenhouse gas emissions (Smith *et al*. 2008). While burning fossil fuels adds carbon to the atmosphere, bioenergy crops, if managed correctly, avoid this by recycling carbon. Although carbon is released to the atmosphere when bioenergy feedstocks are burned, carbon is recaptured during plant growth. The replacement of fossil fuel-generated energy with solar energy captured by photosynthesis has the potential to reduce CO_2_, N_2_O and NO_*x*_ emissions.

However, calculating net emissions from bioenergy is tricky (Searchinger *et al*. 2008). First, management practices used to grow crops and forages for bioenergy production will influence net emissions. Development of appropriate bioenergy systems based on perennial plant species that do not require intensive inputs such as tillage, fertilizers and other agrochemicals have the potential to help offset fossil fuel use in agriculture. Bioenergy systems that rely on annual row crops such as corn are not likely to be as beneficial, and expanding these systems can dramatically reduce the delivery of other ecosystem services like biological pest control (Landis *et al*. 2008). Second, even with the use of perennial species and few inputs, there is significant potential for higher, rather than lower, emissions attributable to bioenergy crops, resulting from land-use change as farmers respond to higher prices and convert forest and grassland to new cropland (Fargione *et al*. 2008; Searchinger *et al*. 2008). The production of bioenergy from waste products, such as crop waste, fall grass harvests from reserve lands, or even municipal waste, could avoid land-use change and result in lower CO_2_ emissions.

## 5. Tradeoffs

Several studies have explicitly analysed possible tradeoffs between the supply of various ecosystem services from agricultural systems. In general, ecosystem services are not independent of one other and the relationships between them are likely to be highly nonlinear. For agriculture, the problem is typically posed as a tradeoff between provisioning services—i.e. production of agricultural goods such as food, fibre or bioenergy—and regulating services such as water purification, soil conservation or carbon sequestration (MEA 2005). Cultural services and biodiversity conservation are also often viewed as tradeoffs with production.

Tradeoffs among ecosystem services should be considered in terms of spatial scale, temporal scale and reversibility (Rodriguez *et al*. 2006). Are the effects of the tradeoff felt locally, for example on-farm, or at a more distant location? How quickly does the tradeoff occur? Are the effects reversible and if so, how quickly can they be reversed? Management decisions often focus on the immediate provision of a commodity or service, at the expense of this same or another ecosystem service at a distant location or in the future. As either the temporal or spatial scale increases, tradeoffs become more uncertain and difficult to manage.

Management is further complicated by biophysical and socioeconomic variation, since every hectare of a given habitat is not of equal value in generating a given ecosystem service (Nelson *et al*. 2009). For natural ecosystems, habitat quality, size of unit and spatial configuration are likely to influence the services provided by the ecosystem. For agroecosystems, management practices, along with access to market and patterns of trade are likely to be critical to the provision of ecosystem services. Furthermore, the values of both market and non-market goods and services will vary according to various biophysical and socioeconomic factors. Without information on the factors that influence the quantity and value of ecosystem services, it is difficult to design policies, incentives or payment schemes that can optimize the delivery of those services (Nelson *et al*. 2009).

Ecosystem services are provided to agriculture at varying scales, and this can influence a farmer's incentives for protecting the ecosystem service. Farmers have a direct interest in managing ecosystem services such as soil fertility, soil retention, pollination and pest control, because they are provided at the field and farm scale. At larger scales, benefits are likely to accrue to others, including other farmers, in addition to the farmer providing the resource. A farmer who restores on-farm habitat complexity increases pollination and pest control services to her neighbours as well as herself. The neighbours benefit from these services without having to give up land that would otherwise produce crops and generate income. Greater landscape complexity may be considered a common pool resource, and a farmer, acting alone, may lack the incentive to set aside the optimal amount of habitat for both the farmer and the neighbour (Zhang *et al*. 2007).

Recent studies suggest that tradeoffs between agricultural production and various ecosystem services are not inevitable and that ‘win–win’ scenarios are possible. An analysis of yields from agroecosystems around the world indicates that, on average, agricultural systems that conserve ecosystem services by using practices like conservation tillage, crop diversification, legume intensification and biological control perform as well as intensive, high-input systems (Badgley *et al*. 2007). The introduction of these types of practices into resource-poor agroecosystems in 57 developing countries resulted in a mean relative yield increase of 79 per cent (Pretty *et al*. 2006). In these examples, there was no evidence that the provisioning services provided by agriculture were jeopardized by modifying the system to improve its ability to provide other ecological services. These analyses suggest that it may be possible to manage agroecosystems to support many ecosystem services while still maintaining or enhancing the provisioning services that agroecosystems were designed to produce. Sustainable intensification will depend on management of ecosystem processes rather than fossil fuel inputs (Baulcombe *et al*. 2009).

Futures scenarios are an increasingly common tool used to evaluate tradeoffs between commodity production, ecosystem services and the conservation of biodiversity in various ecosystems, including agroecosystems (MEA 2005). In addition, advances in spatially explicit modelling have greatly improved the ability to estimate the production of ecosystem services from landscapes. Analysis of the provision of agricultural goods and other ecosystem services in an agricultural valley in Oregon, USA, found few tradeoffs between ecosystem services and biodiversity conservation (Nelson *et al*. 2009). The spatially explicit modelling tool InVEST (integrated valuation of ecosystem services and tradeoffs—Tallis & Polasky 2009) was used to evaluate three stakeholder-defined scenarios of land use through to 2050, including current land-use patterns, increased development or increased conservation. The models predicted changes in commodity production, biodiversity conservation and ecosystem services (hydrological services, soil conservation and carbon sequestration) under the three scenarios. In general, scenarios that scored high on delivering ecosystem services also scored high on biodiversity conservation. Scenarios with increased development had higher commodity values and lower levels of conservation and ecosystem services, but this tradeoff disappeared when payments for carbon sequestration were included. Other spatially explicit studies have also found that biodiversity conservation and carbon sequestration can be achieved in agricultural landscapes (Eigenbrod *et al*. 2009). Clearly, more detailed studies like these are needed to reach a conclusion about the ecological and economic conditions that may lead to tradeoffs between agricultural production and ecosystem services.

### (a) Future trends

Current FAO projections suggest that the rate of conversion of forested land to agriculture will continue to slow through to 2050, there will be little change in grazing area, and protected areas will increase (FAO 2003, 2006). Increases in protected areas will assist in maintaining the flow of ecosystem services like water provisioning, pollination and biological control to agriculture. Advances in sustainable agriculture in developed countries should also lead to enhanced ecosystem services in agricultural landscapes. In some regions, however, conversion of land to urbanization is expected to increase dramatically and will put significant stress on the availability of agricultural land and protected areas. At the global scale, the growth of demand for all crop and livestock products is projected to be lower than in the past: 1.5 per cent per annum in the period 2000–2030 and 0.9 per cent for 2030–2050 when compared with rates around 2.1–2.3% in the preceding four decades, in part due to the lower population growth (FAO 2006).

Despite slowing demand growth, ecosystem disservices are likely to increase as a result of intensification of both crop and animal production, particularly in developing countries where demand for energy-intensive food is expected to grow. The current trend for increasing emissions and water pollution from nitrogen fertilizers with agricultural intensification is forecast to continue through to 2050, despite potential increases in fertilizer-use efficiency (FAO 2003). N-use efficiency is complex and fertilizer prices are likely to remain low. The predicted growth of confinement systems for animal production in developing countries will lead to increased methane and N_2_O emissions from manure, even as improvements in productivity reduce emissions per animal (IPCC 2007). Pesticide use and its non-target effects are likely to increase in some regions through to 2030, while decreasing in others because of increasing regulation and IPM adoption (FAO 2003).

Agricultural intensification is likely to interact with climate change in several ways. Increased frequency of flooding and droughts will increase nutrient losses through runoff and emissions, while over-extraction of groundwater in intensified systems may be exacerbated by drought. At mid- to high latitudes, crop productivity is expected to increase slightly, then decline, with rising temperatures (IPCC 2007). At lower latitudes, productivity is likely to decline even with small temperature increases. Some of the most food-insecure regions, including sub-Saharan Africa, are projected to experience severe declines in agricultural production owing to water shortages by 2020. Moreover, the ability of natural ecosystems to provide ecosystem services to agriculture is expected to be compromised by the interaction of rising temperatures, flooding, drought, pollution and fragmentation (IPCC 2007).

In the face of climate change, resilient agricultural systems with limited fossil fuel inputs will be needed (Lin *et al*. 2008). Sustainable intensification through the management of ecosystem processes has the potential to increase food production while minimizing some of the negative impacts of agricultural intensification on biodiversity and ecosystem services (Baulcombe *et al*. 2009).

## 6. Conclusions

Agricultural systems provide provisioning ecosystem services that are essential to human wellbeing. They also provide and consume a range of other ecosystem services, including regulating services and services that support provisioning. Maximizing provisioning services from agroecosystems can result in tradeoffs with other ecosystem services, but thoughtful management can substantially reduce or even eliminate these tradeoffs. Agricultural management practices are key to realizing the benefits of ecosystem services and reducing disservices from agricultural activities. These challenges will be magnified in the face of climate change, but there have been several recent advances in our ability to estimate the value of various ecosystem services related to agriculture, and to analyse the potential for minimizing tradeoffs and maximizing synergies. Future research will need to tackle these challenges in spatially and temporally explicit frameworks.
